# Biomass Growth and Fatty Acid Production by the Marine *Thraustochytrium* sp. RT2316-16 in Chemically Defined Media

**DOI:** 10.3390/md23120482

**Published:** 2025-12-17

**Authors:** Liset Flores, María Paz Lefiguala, Carolina Shene

**Affiliations:** 1 Department of Chemical Engineering, Center of Food Biotechnology and Bioseparations, BIOREN, and Centre of Biotechnology and Bioengineering (CeBiB), Universidad de La Frontera, Temuco 4780000, Chile; liset.flores@ufrontera.cl; 2 Doctorate in Engineering Sciences with Specialization in Bioprocess, Universidad de La Frontera, Temuco 4780000, Chile; m.lefiguala01@ufromail.cl

**Keywords:** *Thraustochytrium* sp., thraustochytrids, eicosapentaenoic acid, docosahexaenoic acid, vitamin B6, amino acids

## Abstract

The biomass and lipid production responses of the psychrophilic marine thraustochytrid *Thraustochytrium* sp. RT2316-16 were assessed in chemically defined media comprising glucose, up to 17 amino acids and up to 9 B-vitamins and mineral salts. Compared to the control medium with all amino acids and B-vitamins (biomass concentration: 7.1 ± 0.1 g L^−1^; total lipid content: 30.4 ± 0.5% of the DW), the growth of RT2316-16 was reduced by more than 50% in the medium that lacked cyanocobalamin or pyridoxamine. The total lipid content of the biomass grown in the absence of vitamins was 63% lower than in the biomass produced in the control medium. The composition of the B-vitamin mixture modulated the fatty acid composition, an effect that may have been related to the availability of dissolved oxygen. In bioreactor culture with the dissolved oxygen level controlled to ≥10% of air saturation, the microorganism consumed all 17 amino acids; 8 of the amino acids were fully consumed within a 0–33 h period, in which the specific growth rate was 0.065 h^−1^. Under these culture conditions, the sum of eicosapentaenoic acid and docosahexaenoic acid in the total fatty acid content rose from 15% (at time 0) to 54% (after 95 h). A medium that contained the 9 amino acids that were not preferentially consumed favored the accumulation of total lipids, but reduced biomass growth.

## 1. Introduction

*Thraustochytrium* sp. RT2316-16 is a psychrophilic marine thraustochytrid. This aerobic heterotroph produces triglycerides that are rich in long-chain polyunsaturated fatty acids such as eicosapentaenoic acid (EPA) and docosahexaenoic acid (DHA) [1]. RT2316-16 also produces carotenoids [2,3], the co-enzyme Q_10_ [4,5], and squalene [6]. The quantities of the above-mentioned metabolites in the biomass are affected by the composition of the culture medium and the culture conditions. Media for culturing thraustochytrids typically contain a carbon source, a complex nitrogen source, vitamins, and minerals. Commonly used carbon sources are glucose and glycerol [7,8], as they permit rapid growth of the biomass and promote the accumulation of lipids under suitable conditions. Yeast extract and peptones are commonly used nitrogen sources [9,10]. These nitrogen sources are poorly defined, containing a complex mixture of amino acids, other nitrogenous compounds, and carbohydrates such as glucose, trehalose, and glycogen [11]. In addition, yeast extract contains nucleotides, B-group vitamins, and minerals. The exact composition of the yeast extract depends on the specific yeast used and the method of production [12].

Growth, protein synthesis, and other aspects of metabolism in heterotrophs are generally enhanced if a suitable complement of amino acids is provided in the culture medium [13]. The requirements of specific amino acids for thraustochytrids have not been established, although some species thrive in media formulated exclusively with inorganic nitrogen [14] or glutamate [15]. This suggests an ability to synthesize the necessary amino acids. This notwithstanding, a supply of some amino acids in the culture medium may greatly improve the culture performance relative to media that provide only inorganic nitrogen [1].

Although carbon and nitrogen nutrients provide the main elements needed to generate microbial biomass, many other trace elements (e.g., P, S, and Fe) and some complex organic molecules (e.g., some vitamins) are typically needed. Vitamins function as cofactors of enzymes and participate in metabolic processes for other essential roles. Some thraustochytrids have been shown to require an external supply of certain vitamins. For example, thiamine (vitamin B1) has been identified as a unique essential vitamin for *Auranthiochytrium* sp. [16] and vitamin auxotrophy for thiamine and cyanocobalamin (B12) has been reported in *Thraustochytrium aureum*, *Thraustochytrium roseum*, and *Schizochytrium aggregatum* [17]. Other thraustochytrids have been shown to take up vitamin B12 from the culture medium and B12 has been found to influence the composition of the fatty acids in the cell [18]. Mineral salts provide essential trace metals and other elements such as P and S. Phosphorous is required for the synthesis of nucleic acids and phospholipids. Metal (Fe, Zn, Cu, Co, and Mn) ions often function as cofactors in essential enzymes.

The elemental composition of thraustochytrid biomass may be a useful guide for designing media to culture them, although the composition is likely to vary somewhat with the species. The stage of growth, whether the exponential growth phase or the stationary phase, also affects the biomass composition [19]. Typically, thraustochytrid biomass has the following elemental composition: 64.0–67.5% *ww*^−1^ C, 9.9–13.2% *ww*^−1^ H, 1.9 to 2.2% *ww*^−1^ N, and less than 1% *ww*^−1^ S [19].

In view of their potential impact on key aspects of thraustochytrid metabolism, the present work focused on elucidating the effects of the mineral salts and B-group vitamins that have previously been used for growing *Thraustochytrium* sp. RT2316-16 [1,2,3,4,5,6], but without an understanding of whether or how they impacted the growth and production of metabolites such as fatty acids. As noted above, the interest in RT2316-16 is driven by its ability to produce metabolites of commercial interest [2,3,4,5,6]. The culture media previously used for growing RT2316-16 comprises a carbon source (typically glucose or glycerol) and some organic nitrogen sources (typically yeast extract and monosodium glutamate). Yeast extract is a poorly defined mixture that contains nearly all the main amino acids, many nitrogen-containing vitamins, and nucleotides. To avoid uncertainties, the present work used chemically defined media comprising pure compounds including individual vitamins and up to 17 amino acids. The growth of RT2316-16 in chemically defined media was evaluated initially in Erlenmeyer flasks and subsequently selected media were further evaluated in a bioreactor in which the dissolved oxygen concentration was controlled. The information generated is expected to guide the design of media for producing lipid-rich biomass of RT2316-16 and for influencing the composition of the lipids in the biomass so that fatty acids of particular interest can be overproduced.

## 2. Results

### 2.1. Biomass Growth and Lipid Production

**Figure 1:**
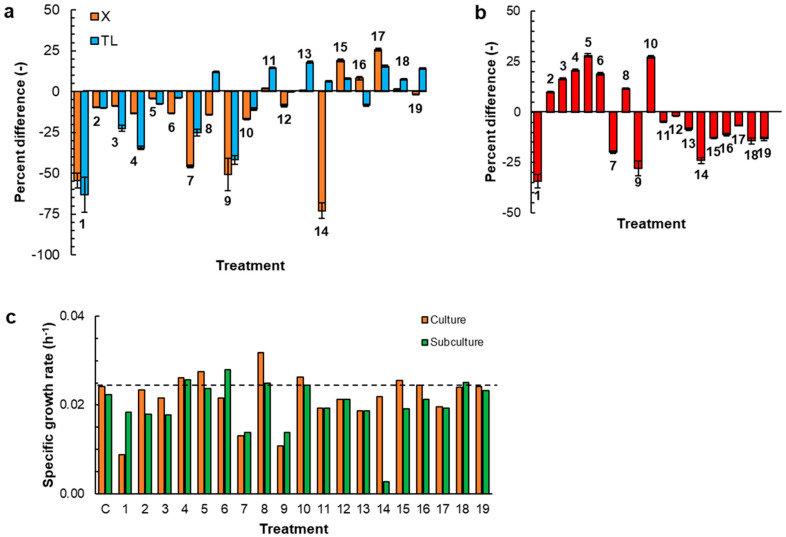
The percentage difference in the following, relative to the control (C) medium: (**a**) biomass concentration (X) and total lipid content (TL) in the biomass at 168 h; (**b**) residual glucose at 168 h; and (**c**) specific growth rate (0–48 h). The treatments were as follows: (1) C without vitamins; (2) C without thiamine; (3) C without Ca-pantothenate; (4) C without nicotinic acid; (5) C without pyridoxine; (6) C without biotin; (7) C without cyanocobalamin; (8) C without riboflavin; (9) C without pyridoxamine; (10) C without pABA; (11) C with pyridoxine (0.72 mg L^−1^) as the only B6 vitamer; (12) C with pyridoxamine (0.72 mg L^−1^) as the only B6 vitamer; (13) C with pyridoxal 5′-phosphate (0.72 mg L^−1^) as the only B6 vitamer; (14) C without S_1_ (KH_2_PO_4_) and S_2_ (MnCl_2_ 4H_2_O, ZnSO_4_ 7H_2_O, CoCl_2_ 6H_2_O, CuSO_4_ 5H_2_O, NiSO_4_ 6H_2_O, and FeSO_4_ 7H_2_O) (Tables S1 and S2); (15) C with S_1_ at 2× and S_2_; (16) C with S_1_ and S_2_ at 2×; (17) C with S_1_ at 2× and S_2_ at 2×; (18) C with trehalose at 1 g L^−1^; and (19) C with trehalose at 0.5 g L^−1^.

The vitamins in the control medium (Table S1; Supplementary Materials) were provided at concentrations that were previously found to support good growth and glucose uptake by RT2316-16 [1,3], although no information existed on the possible effects of these vitamins individually, or their total absence. The effects of the absence of individual vitamins on the culture characteristics are shown as Treatments 1–10 in Figure 1a. The effects are shown as a percentage difference relative to the control. All data are for an incubation period of 168 h, i.e., the end of the batch culture. The time profiles of the concentrations of biomass and glucose in all the Treatments are shown in Figure S1 (Supplementary Materials).

Compared with the control medium (biomass concentration: 7.1 ± 0.1 g L^−1^ (Figure S1, Supplementary Materials); total lipid content: 30.4 ± 0.5% of the DW), the medium without the vitamins produced 54% less biomass and this biomass had a total lipid content that was 63% lower than in the biomass grown in the control medium (Treatment 1; Figure 1a). A lack of any of the nine B-vitamins (Treatments 1–10; Figure 1a) reduced the final biomass concentration relative to the control. This concentration-reducing effect was weakest (a 4% lower biomass concentration relative to the control) if the missing vitamin was the B6 vitamer pyridoxine (Treatment 5; Figure 1a). If the missing B6 vitamer was pyridoxamine (Treatment 9; Figure 1a), the decrease in the final biomass concentration relative to the control was not significantly different (*p* > 0.05) to the reduction observed in the vitamin-free medium (Treatment 1; Figure 1a). If the medium lacked cyanocobalamin (Treatment 7; Figure 1a), the final biomass concentration was nearly the same as in the vitamin-free medium.

The medium that lacked riboflavin was the only one that produced biomass with a total lipid content that exceeded the lipid content of the biomass grown in the control medium (Treatment 8; Figure 1a). The glucose was not fully consumed in all of the cultures (Figure S1 in Supplementary Materials; Figure 1b). The glucose consumption was roughly directly proportional to the amount of biomass that was produced in each treatment (Treatments 1, 7, and 9; Figure 1b).

The biomass in the 168 h cultures was further subcultured (Section 2.2) in the same medium to evaluate if the media used to grow the inoculum had an effect on the growth of RT2316-16. Cells cultivated in a rich medium such as GYM (used to grow the inoculum of the first cultures) might store vitamins and other cofactors available in the yeast extract, favoring their initial growth in the chemically defined medium. This subculturing was carried out for all treatments. The growth curves and residual glucose concentration profiles of the initial culture and subsequent subculture are compared in Figure S1 (Supplementary Materials) for all treatments. The specific growth rates were calculated for the 0–48 h period (the period of rapid growth) (Figure S1; Supplementary Materials), both for the initial culture and the subculture. The results are shown in Figure 1c. The biomass subcultured in the vitamin-free medium (Treatment 1; Figure 1c) grew at a specific growth rate that was 2-fold higher than the corresponding growth rate in the first stage culture (9 × 10^−3^ h^−1^). On the other hand, the specific growth rate of the biomass subcultured in the medium that lacked riboflavin (Treatment 8; Figure 1c) was ~20% lower than the specific growth rate in the first stage culture. It should be noted that in the first culture RT2316-16 grew at the highest specific growth rate (0.032 h^−1^) in the riboflavin-free medium (Treatment 8; Figure 1c).

Vitamin B6 is not a single entity but a group of six different compounds or vitamers. The control medium contained two of these vitamers at different concentrations: pyridoxine at 0.029 mg L^−1^ and pyridoxamine at 0.72 mg L^−1^ (Table S1 in Supplementary Materials). While the omission of pyridoxine had a relatively small effect on the biomass growth and total lipids in the biomass (Treatments 5; Figure 1a), the omission of pyridoxamine from the culture medium exerted the most important effect among the treatments (Treatments 9; Figure 1a).

The effects of three of the key vitamers (i.e., pyridoxine (B6-1), pyridoxamine (B6-2), and pyridoxal 5′-phosphate (B6-3)) on the culture were evaluated. The media were formulated with the other seven B-vitamins (Table S1, Supplementary Materials) and a single B6 vitamer, either pyridoxine (0.72 mg L^−1^; Treatment 11), pyridoxamine (0.72 mg L^−1^; Treatment 12), or pyridoxal 5′-phosphate (0.72 mg L^−1^; Treatment 13). Time profiles of biomass and glucose concentrations in initial cultures and the subcultures of Treatments 11–13 are shown in Figure S2 (Supplementary Materials). Irrespective of the B6 vitamer present in the culture medium, the percentage difference in biomass concentration relative to the control was <10% (Treatments 11–13; Figure 1a). The specific growth rate of RT2316-16 was not affected by the B6 vitamer supplied and the initial culture and the corresponding subculture had identical specific growth rates (Treatments 11–13; Figure 1c). However, relative to the control medium, the media supplied with only pyridoxine, or only pyridoxal 5′-phosphate, resulted in biomass that was 14–18% richer in total lipids (Treatments 11–13; Figure 1a).

In the next set of experiments, the effect of two groups of salts (KH_2_PO_4_ (group S_1_); or MnCl_2_ 4H_2_O, ZnSO_4_ 7H_2_O, CoCl_2_ 6H_2_O, CuSO_4_ 5H_2_O, NiSO_4_ 6H_2_O, and FeSO_4_ 7H_2_O (group S_2_)) supplied to attain the specified concentrations were assessed. The concentrations of the other components in the media were identical to those in the control medium (Table S1; Supplementary Materials). Time profiles of the concentrations of biomass and glucose in the various cultures are shown in Figure S3 (Supplementary Materials). Compared with the control medium, the medium that lacked all mineral salts produced a biomass concentration that was 73% lower (Treatment 14, Figure 1a). In contrast, the culture media that included either phosphate, or phosphate and all the other mineral salts at levels 2-fold higher than the level in the control medium, resulted in biomass concentrations that were 19 and 26% higher than the control (Treatments 15 and 17; Figure 1a), respectively. The biomass produced in these media had total lipid contents that were 8–16% higher compared with the control (Treatments 15 and 17; Figure 1a).

The disaccharide trehalose is commonly found in yeast extract. Therefore, its effect on the growth of RT2316-16 was tested at trehalose concentrations of 1 and 0.5 g L^−1^ (Treatments 18 and 19, respectively; Figure 1). At these concentrations, trehalose barely affected the final biomass concentration (a change of less than 2%; Treatments 18 and 19, Figure 1a), but it did increase the total lipid content of the biomass (an 8% increase in Treatment 18 and a 14% increase in Treatment 19; Figure 1a) relative to the biomass produced in the control medium. The trehalose-containing media had less residual glucose (Treatments 18 and 19; Figure 1b), suggesting that trehalose enhanced glucose uptake. The specific growth rate of the biomass was not affected by trehalose compared to the control at the concentrations used (Treatments 18 and 19; Figure 1c).

### 2.2. Fatty Acid Content of the Biomass and Fatty Acid Profile of the Total Lipids

**Figure 2:**
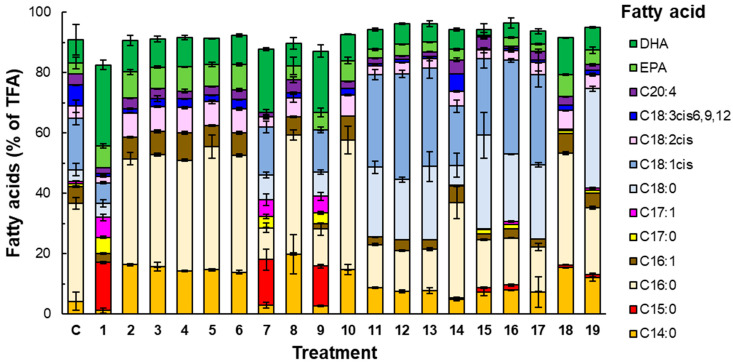
The composition of the fatty acids (by percentage of the total fatty acids, TFA) of RT2316-16 grown (for 168 h) in different media, or treatments. The treatments were as follows: (1) the control medium C without vitamins; (2) C without thiamine; (3) C without Ca-pantothenate; (4) C without nicotinic acid; (5) C without pyridoxine; (6) C without biotin; (7) C without cyanocobalamin; (8) C without riboflavin; (9) C without pyridoxamine; (10) C without pABA; (11) C with pyridoxine (0.72 mg L^−1^) as the only B6 vitamer; (12) C with pyridoxamine (0.72 mg L^−1^) as the only B6 vitamer; (13) C with pyridoxal 5′-phosphate (0.72 mg L^−1^) as the only B6 vitamer; (14) C without S_1_ (KH_2_PO_4_) and S_2_ (MnCl_2_ 4H_2_O, ZnSO_4_ 7H_2_O, CoCl_2_ 6H_2_O, CuSO_4_ 5H_2_O, NiSO_4_ 6H_2_O, and FeSO_4_ 7H_2_O) (Table S1); (15) C with S1 at 2× and S_2_; (16) C with S_1_ and S_2_ at 2×; (17) C with S_1_ at 2× and S_2_ at 2×; (18) C with trehalose at 1 g L^−1^; and (19) C with trehalose at 0.5 g L^−1^.

The composition of the fatty acids in the total lipid content of the biomass grown in various media after 168 h of batch culture is shown in Figure 2. The fatty acid composition depended on the specific vitamins provided in the culture media (Figure 2). Media that lacked certain vitamins individually (i.e., thiamine, Ca-pantothenate, nicotinic acid, pyridoxine, biotin, riboflavin, or pABA; Treatments 2–6, 8, and 10 in Figure 2) produced biomass with myristic acid and palmitic acid as the main fatty acids (on average of 54% of the total fatty acids). The vitamin-free medium (Treatment 1; Figure 2) and the media that lacked either cyanocobalamin (Treatment 7) or pyridoxamine (Treatment 9) generated biomass that was characterized by the presence of stearic acid, oleic acid, and the odd-chain fatty acids pentadecanoic acid and heptadecanoic acid (Figure 2). The percentage of saturated fatty acids was higher in Treatments 2–6 and 8, compared to Treatments 1, 7, and 9 (Figure 2). In contrast, the percentage of DHA was higher in Treatments 1, 7, and 9 compared to Treatments 2–6 and 8 (Figure 2). The biomass grown in the control medium (Treatment C; Figure 2) had a fatty acid profile that was similar to the profile of the biomass produced in Treatments 1, 7, and 9, although no odd-chain fatty acids were detected (Figure 2).

The different B6 vitamers had no effect on the fatty acid composition (Treatments 11–13; Figure 2). In terms of the inorganic salts, the biomass grown in the medium that was free of KH_2_PO_4_ and other salts (i.e., Treatment 14), produced lipids with a fatty acid composition similar to that of the lipids from the biomass produced in the control medium. Stearic acid and oleic acid were not found in the biomass produced in the medium that was supplemented with trehalose at a concentration of 1 g L^−1^ (Treatment 18; Figure 2), but stearic acid was found in the biomass grown with a trehalose concentration of 0.5 g L^−1^ (Treatment 19; Figure 2).

### 2.3. Bioreactor Culture

**Figure 3:**
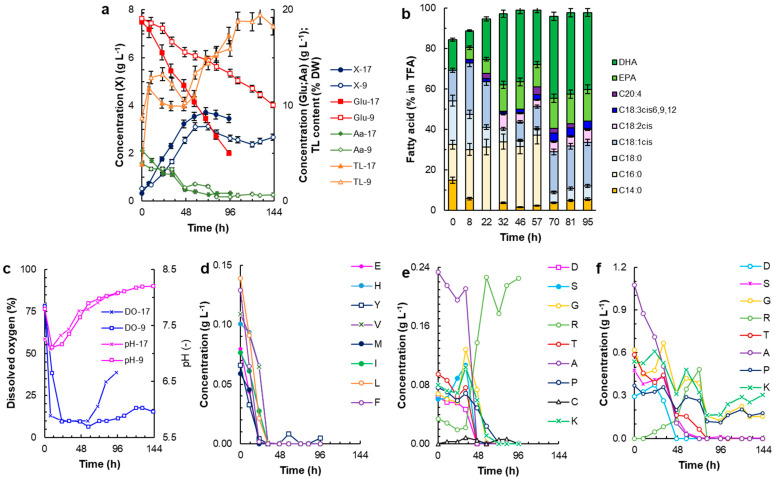
The time profiles of the RT2316-16 culture in the bioreactor: (**a**) the concentration of biomass (X), glucose (Glu), and total amino acids (Aa) in chemically defined media with either 17 amino acids (X-17; Glu-17; Aa-17) or 9 amino acids (X-9; Glu-9; Aa-9); (**b**) composition profiles of the total fatty acid content in the biomass produced by various time durations in the medium with 17 amino acids; (**c**) changes in pH and dissolved oxygen (DO) concentration in the medium with 17 (pH-17; DO-17) amino acids and 9 (pH-9; DO-9) amino acids; (**d**,**e**) concentrations of specific amino acids in the medium with 17 amino acids; and (**f**) concentrations of specific amino acids in the medium with 9 amino acids. The amino acids are identified by the following standard codes: glutamate (E); histidine (H); tyrosine (Y); valine (V); methionine (M); isoleucine (I); leucine (L); phenylalanine (F); aspartate (D); serine (S); glycine (G); arginine (R); threonine (T); alanine (A); proline (P); cysteine (C); and lysine (K).

*Thraustochytrium* sp. RT2316-16 was grown in a bioreactor (Section 4.3) with the dissolved oxygen (DO) level controlled to a level that equaled or exceeded 10% of the air saturation. Different experiments used both the control medium and the medium CD9 (Tables S1 and S3; Supplementary Materials). These media differed only in the numbers and concentrations of the amino acids supplied (Table S3; Supplementary Materials); they were identical in terms of the vitamins and the mineral salts provided (Table S1; Supplementary Materials). The relevant culture profiles are shown in Figure 3.

The biomass production was distinctly higher in the control medium than in medium CD9 (Figure 3a). The maximum biomass concentration in the control medium was 3.7 g L^−1^ at 70 h (Figure 3a). This peak biomass concentration was 47% lower than the concentration obtained in Erlenmeyer flasks in the control medium (7.1 ± 0.1 g L^−1^ at 168 h; Figure S1 in Supplementary Materials). Nonetheless, in the bioreactor the biomass grew at a specific growth rate of 0.062 h^−1^ (0–32 h) that was higher than in the Erlenmeyer flasks (Figure 1c). The biomass produced in the control medium had a total lipid content of 17% *ww*^−1^ at 95 h, i.e., 44% lower than the biomass grown in the Erlenmeyer flasks. The growth in the control medium in the bioreactor ceased around 70 h, although the medium still had plenty of residual glucose but was nearing exhaustion of the amino acids (Figure 3a,d,e). The concentration of dissolved oxygen declined rapidly during the first 6–24 h, depending on the medium, because of consumption by the cells during rapid growth (Figure 3c). The DO remained relatively stable at ≥10% of the air saturation for ~57 h during growth, but rose afterwards as the consumption by the cells slowed. Around 57 h, the growth began to slow in both media, although the DO level in CD9 was much lower than in the control medium until the end of the culture (Figure 3c). This suggests that oxygen was being consumed at right up to 144 h in the CD9 culture (Figure 3c). In both media, the rate of consumption of glucose was faster during the period of rapid growth compared to the later period (Figure 3a). Around 32 h, the rate at which glucose was consumed by the biomass cultivated in the control medium slowed, corresponding to a point in time by which 8 (glutamate, histidine, tyrosine, valine, methionine, isoleucine, leucine, and phenylalanine) of the 17 amino acids were totally consumed (Figure 3d). Eight of the remaining amino acids were exhausted between 48 h and 73 h, whereas the concentration of residual arginine began to rise around 33 h (Figure 3e). In both media, there was a rapid initial decline in pH, but from ~12 h the pH rose from around 7.3 to up to 8.1 depending on the culture medium (Figure 3c). In both media, both during rapid growth and afterwards, the total lipid content of the biomass increased with time (Figure 3a); however, the rate of increase in total lipids content varied with the phase of growth. The composition of the fatty acids in the total lipids produced by the biomass cultivated in the control medium also depended on the phase of growth (Figure 3b). Major changes in fatty acid composition were seen between 57 and 70 h when palmitic acid disappeared and the percentage of oleic acid increased (Figure 3b). After this time point, the percentages of DHA and EPA in the total fatty acids remained above 38% and 15%, respectively (Figure 3b).

RT2316-16 was cultured in the medium CD9, which contained only the nine amino acids that were late-consumed (Figure 3e), to determine if the eight amino acids that were consumed preferentially inhibited the uptake of these nine amino acids. Although the total amino acid concentration in medium CD9 was the same as that in the control medium (5 g L^−1^) (Table S3; Supplementary Materials), the maximum biomass concentration attained in medium CD9 was 16% lower than the concentration obtained in the control medium (Figure 3a) and the specific growth rate was 0.035 h^−1^ (0–58 h). In medium CD9, glucose was consumed at a much slower rate than in the control medium (Figure 3a). For example, in medium CD9, the residual glucose concentration at 96 h was 13 g L^−1^, whereas in the control medium it was 5 g L^−1^ (Figure 3a). In medium CD9, glycine, lysine, and proline were not fully consumed by the end of the culture (144 h); however, the other 6 amino acids were fully consumed by ~58 h when the growth ceased (Figure 3f).

## 3. Discussion

Media for culturing thraustochytrids generally contain complex organic nitrogen sources such as yeast extract and/or peptones that provide amino acids, peptides, vitamins, and other unknown nutritional factors. These culture media often support rapid growth and allow high concentrations of biomass to be attained. However, the composition of the complex nitrogen sources varies depending on the method of production used by a commercial supplier. Consequently, the use of these nitrogen sources in culture media results in variable performance. More consistent and predictable performance of a culture can be obtained by using media that are fully chemically defined, that is, the identity and the concentration of each chemical entity in the medium is known. Chemically defined media may be easily and consistently formulated to direct microbial metabolism to preferentially produce the metabolites of interest, while suppressing the wasteful diversion of the media nutrients for the production of unwanted metabolites. This is because the flux through metabolic pathways is determined by the availability of specific nutrients, precursors, stimulants, and suppressors in the culture medium. All media used in the present work were fully chemically defined. The chemical identities and initial concentrations of all the components used in the media are shown in Tables S1 and S3 (Supplementary Materials).

The B-group vitamins are known cofactors in the metabolism of amino acids and fatty acids and are also involved in the generation of biochemical energy in diverse microbes [20,21,22,23,24]. These vitamins likely served similar roles within RT2316-16. Studies in thraustochytrids have confirmed the metabolic stimulatory effects of various B-vitamins supplied in culture media [16,25]. Work in media that lacked specified vitamins individually revealed that each of the nine B-vitamins (thiamine, Ca-pantothenate, pyridoxine, biotin, cyanocobalamin, riboflavin, pyridoxamine, nicotinic acid, and p-aminobenzoic acid) affected biomass production. A chemically defined medium that lacked either cyanocobalamin (vitamin B12) or pyridoxamine (vitamin B6) was equivalent in biomass production performance to a medium devoid of vitamins (Figure 1a). Not all microorganisms are able to synthesize the active form of vitamin B6 (pyridoxal 5′-phosphate) and thus they depend on its uptake or use the salvage pathways to convert the B6 vitamers available in the culture medium to the active form [26]. In the salvage pathways, pyridoxine and pyridoxamine are converted into the active form of vitamin B6 through reactions catalyzed by pyridoxal-kinases and pyridoxal-oxidases. Kinases with different substrate preferences have been described [27]. Differences in biomass growth in different media containing either pyridoxine or pyridoxamine suggested that pyridoxine supplied at a concentration of 0.029 g L^−1^ could not replace the effect of pyridoxamine supplied at a level of 0.72 mg L^−1^. Nonetheless, the biomass concentrations in media containing either pyridoxine, pyridoxamine, or pyridoxal 5′-phosphate at a concentration of 0.72 m L^−1^ differed little (<10% difference), although the biomass grown in media with pyridoxine or pyridoxal 5′-phosphate had on average more lipids (15–18% more) than the biomass produced in a pyridoxamine-containing medium.

Cyanocobalamin (vitamin B12) is a cofactor of methionine synthase. The amino acid methionine is required for protein synthesis and has other metabolic roles. Cyanocobalamin is also a cofactor of methylmalonyl-CoA mutase, an enzyme involved in the conversion of propionyl-CoA, which is produced in the metabolism of valine, methionine, isoleucine, and threonine, and in the β-oxidation of odd-chain fatty acids, to succinyl-CoA that is fed into the tricarboxylic acid cycle. If conversion of propionyl-CoA to succinyl-CoA is insufficient, less energy is produced due to the oxidation of certain amino acids and the available propionyl-CoA may be used as the starting substrate to produce odd-chain fatty acids by the action of fatty acid synthase. In the present study, the medium lacking cyanocobalamin favored the accumulation of odd-chain fatty acids. The results also showed that odd-chain fatty acids accumulated when RT2316-16 was grown in a medium that lacked pyridoxamine. Thus, the accumulation of propionyl-CoA, the starting substrate in the biosynthesis of odd-chain fatty acids in RT2316-16, and its occurrence in media deficient in cyanocobalamin and pyridoxamine are probably related through an unknown mechanism.

Deficiency of either cyanocobalamin or pyridoxamine decreased biomass growth. The two vitamins are cofactors of different reactions in the homocysteine metabolism. The amino acid homocysteine accumulates in the absence of cyanocobalamin and also in the absence of pyridoxal 5′-phosphate. Homocysteine has a growth inhibitory effect in bacteria such as *Escherichia coli* because of its interference with the synthesis of isoleucine [28]. In the present study, isoleucine was one of the amino acids that was rapidly and preferentially consumed by RT2316-16 and this likely explained the initial growth in the medium that was free of cyanocobalamin or pyridoxamine (Figure S1) and the subsequent lack of growth might be due to isoleucine exhaustion. In *E. coli* and *Saccharomyces cerevisiae,* homocysteine is converted into homocysteine-thiolactone, preventing its incorporation into tRNA and protein [29]. This reaction consumes ATP so that less energy is available for protein synthesis and growth [30]. Consistent with this mechanism, the specific growth rates of RT2316-16 were among the lowest in media that lacked either cyanocobalamin or pyridoxamine (Figure 1c).

Riboflavin was the only B-vitamin that when it was not supplied had a negative effect on the production of biomass, but increased the total lipid content of the biomass. Riboflavin is involved in the metabolism of amino acids and the β-oxidation of fatty acids [31]. An impaired fatty acid oxidation in a riboflavin-deficient medium may have contributed to the observed increase in total lipids in the biomass grown in the relevant medium. However, since riboflavin is used in the synthesis flavin mononucleotide (FMN) and flavin adenine dinucleotide (FAD) cofactors of a multitude of enzymes in several metabolic pathways (β-oxidation, respiratory chain, amino acids synthesis, metabolism of vitamins, glutathione metabolism, among others) and due to the relatively small effect on biomass growth (14% lower than in the control medium) in a medium deficient in riboflavin it is plausible that RT2316-16 is able to synthesize this vitamin.

An increased phosphate concentration in the medium, or its supplementation with trehalose, enhanced the final biomass concentration relative to the control (Figure 1a). Trehalose is produced by certain microorganisms as a storage carbohydrate and it is known to confer protection against different forms of stress (heat shock, cold shock, and desiccation) [32,33] and has antioxidant activity [34]. Yeast extracts typically used for formulating complex culture media contain trehalose in quantities that depend on the yeast and the growth conditions used in producing it [11]. The small positive effect of trehalose on biomass growth was observed at its higher concentration (1 g L^−1^; Figure 1a), but at a lower concentration (0.5 g L^−1^) the effect was slightly negative. Above a threshold concentration, trehalose may have had a protective effect on RT2316-16, or it may have served simply as an additional source of carbon.

The relative proportion of EPA and DHA in the total fatty acid content of RT2316-16 was affected by some B-vitamins. This effect may have been due to specific vitamins affecting the activity of the enzymes involved in fatty acid synthesis, or it may have been a consequence of the vitamins affecting the fatty acid composition indirectly by affecting the growth rate of the biomass. Culture media that depressed biomass production also reduced the total lipid content of the biomass (lipid content reduced to less than 20% *ww*^−1^ of dry biomass); however, the proportion of EPA and DHA in the total lipid content increased compared to the biomass that had been produced in media that supported lipid synthesis. Although thraustochytrids are obligate aerobes, they can use an oxygen-dependent aerobic pathway, as well as an oxygen-independent pathway, for synthesizing long-chain polyunsaturated fatty acids [35]. The oxygen-dependent pathway requires molecular oxygen for the desaturation of fatty acids. In the shake flask experiments, the dissolved oxygen concentration tended to be low in media that promoted rapid growth because of its faster consumption. Under such conditions, fatty acid desaturation via the aerobic pathway may have been depressed.

Important changes in the composition of the fatty acids produced due to changes in the growth medium composition have been reported in other thraustochytrid strains. The main fatty acids in the strain F24-2 were C16:0, DHA, and n-6 docosapentaenoic acid (DPA); the percentages of these three fatty acids in the total fatty acid content varied in the range of 15.6 to 58.3%, 17.0 to 45.1%, and 9.3 to 23.2%, respectively, when the strain was cultivated in media that differed in the concentration of glucose (5 or 20 g L^−1^), soy peptone (2 or 20 g L^−1^), sea salts (9 or 18 g L^−1^), ammonium sulfate (0.2 or 2 g L^−1^), vitamin B stock solution (0.01 g L^−1^ vitamin B12, 0.01 g L^−1^ biotin, and 2 g L^−1^ thiamine hydrochloride) (1 or 3 mL L^−1^), initial pH (4 or 7), and temperature (25 or 31 °C) [10]. Long-chain polyunsaturated fatty acids (>18:3) were absent from the total lipids of *Aurantiochytrium* sp. T66 when it was cultivated with different volatile fatty acids (formic acid, acetic acid, propionic acid, butyric acid, caproic acid, and valeric acid) as carbon sources at a concentration equal to 2 g L^−1^ [36]; however, at a higher concentration (10 g L^−1^) of the various carbon sources, the percentages of DPA and DHA in the total lipids were 7.5 and 9.6%; 18.3 and 28.8%; 7.3 and 7.3%; 8.9 and 18.6%; 4.9 and 7.9%; and 2.4 and 6.0%, respectively.

The subculture experiments showed that the growth of RT2316-16 in the chemically defined media and the consumption of glucose were not affected by the medium composition used to grow the inoculum (GYM or whether the chemically defined media had different vitamins and/or salt composition). It was shown that *Aurantiochytrium mangrovei* 18W-13a has the capability to store vitamins, enabling growth when it is transferred to a vitamin-free medium until vitamin storage becomes depleted [11].

The observed preferential consumption of certain amino acids by RT2316-16 (Figure 3d–f) has also been reported in other unrelated microorganisms [37]. Further studies are needed to establish the conditions that promote and constrain the cellular uptake of specific amino acids and to elucidate how the differential uptake affects the synthesis of long-chain polyunsaturated fatty acids, carotenoids, and squalene in RT2316-16. The residual concentration of arginine (Figure 3e,f) was found to increase after the peak biomass concentration had been attained and the residual concentrations of the other amino acids had declined to less than 0.19 g L^−1^ (Figure 3e,f). Arginine is the most nitrogen-rich and the most basic of the amino acids. In plants, de novo biosynthesis of arginine occurs in response to abiotic stresses that promote the production of ammonia [38]. Intracellular accumulation of large pools of arginine within vacuoles is known to occur in unrelated microorganisms such as the fungus *Neurospora crassa* [39] and vacuoles have been observed in some thraustochytrids [40].

For commercially useful production of metabolites, the ability to culture a target microorganism to a high concentration in large bioreactors is important. Stirred tank bioreactors are the most used bioreactors for aerobic cultures, including commercial culture of some thraustochytrids [35]. Therefore, RT2316-16 was cultivated in a stirred bioreactor with the dissolved oxygen concentration controlled to ≥10% of the air saturation with a chemically defined medium. These operational conditions are suggested for attaining high percentages of EPA and DHA in the total fatty acid content of RT2316-16. Unexpectedly, the biomass concentration was ~53% lower compared to the concentration that could be attained in the same medium in Erlenmeyer flasks (~7.0 g L^−1^). The substantially lower maximum biomass concentration in the bioreactor was due to the rapid exhaustion of the amino acids because of the high specific growth rate (nearly 2-fold higher than that of the biomass in the Erlenmeyer flasks); after the amino acids were exhausted the total lipid content of the biomass increased (Figure 3a). However, the lipid content of the biomass cultivated in the bioreactor was lower than the lipid content of the biomass cultivated in shake flasks and this explains, in part, the lower biomass concentration in the bioreactor.

The results of this work showed that when *Thraustochytrium* sp. RT2316-16 was cultivated with a mixture of 17 amino acids, 8 of them (glutamate, histidine, tyrosine, valine, methionine, isoleucine, leucine, and phenylalanine) were rapidly consumed. The consumption rate of the other 9 amino acids (aspartate, serine, glycine, arginine, threonine, alanine, proline, cysteine, and lysine) did not increase when the rapidly consumed amino acids were not supplied (medium CD9). The biomass growth and glucose consumption rates in the medium CD9 were significantly reduced, even though the total amino acid concentration was the same (5 g L^−1^). Although RT2316-16 is able to grow with only one amino acid as the sole source of nitrogen [1,4,5], the biomass production depends on the amino acid. It is possible that under the growth conditions tested the biosynthesis of one (or more) of the 8 amino acids that are not found in the medium CD9 diverts intermediates and energy. As a reference, in bacteria, the cost in terms of energy phosphate bonds per molecule of amino acid has been estimated to vary from 11.7 (alanine, glycine, and serine) to 74 (tryptophane) [41].

## 4. Materials and Methods

### 4.1. Microorganism and Inoculum Preparation

The psychrophilic marine protist *Thraustochytrium* sp. RT2316-16 isolated in earlier work [3] was used throughout in aseptic cultures. Pure stock cultures were maintained at 4 °C.

A glucose–yeast extract medium (GYM) with the following composition (g L^−1^) was used to prepare the first inoculum for all culture experiments: glucose (Merck, Darmstadt, Germany) 20, yeast extract (Merck) 6, and monosodium glutamate (Merck) 0.6. The medium was made in artificial seawater (ASW) [42] mixed with distilled water in a volume ratio of 1:1. This medium was supplemented with two vitamin solutions (3.6 mL L^−1^ each) and a solution of mineral salts (24 mL L^−1^). All vitamins were obtained from Sigma-Aldrich (St. Louis, MO, USA) and the mineral salts from Merck. The compositions of these three solutions are shown in Table S2 (Supplementary Materials) and the final concentrations of vitamins and mineral salts in the culture medium are provided in Table S1 (Supplementary Materials). The vitamin and mineral salt solutions were sterilized by being passed through a 0.2 μm sterile membrane filter.

A control medium with the following composition was used (g L^−1^ in 1:1 by volume mixture of ASW and distilled water): glucose 20, total amino acids 5, the two vitamin solutions (3.6 mL L^−1^ each), and the mineral salt solution (24 mL L^−1^). The specific amino acids and their concentrations are shown in Table S3 (Supplementary Materials). All the amino acids were obtained from Sigma-Aldrich. The amino acids and their final concentrations in the media were chosen to match those of a medium formulated with a typical yeast extract [12].

The first inoculum was prepared by transferring 5 mL of a stock culture to 100 mL (250 mL Erlenmeyer flask) of sterile GYM. After incubation (15 ± 1 °C, 4 days, 150 rpm) in an orbital shaker (floor model incubator, model ZHWY-C211D, Zhicheng^®^, Shanghai, China), 10 mL of this first inoculum was used to generate a second inoculum by transferring to 300 mL (500 mL Erlenmeyer flask) of sterile GYM. After incubation as above, a 10 mL aliquot of the second inoculum was aseptically withdrawn and centrifuged (3220 *g*, 10 min, 4 °C) to recover the cell pellet. The pellet was washed in 10 mL of sterile and diluted ASW (1:1 dilution with distilled water) and resuspended in 10 mL of the same sterile fluid for use as inoculum to the treatment cultures. The biomass concentration in the second culture was 6.0 ± 0.5 g L^−1^. Thus, the initial biomass concentration in the culture assays was 0.20 ± 0.02 g L^−1^.

### 4.2. Evaluation of the Effect of Vitamins, Mineral Salts and Trehalose

Four sets of experiments were performed in Erlenmeyer flasks to evaluate the effects of the vitamins, the mineral salts, and the sugar trehalose (C_12_H_22_O_11_, a disaccharide comprising two glucose units). In the first set of experiments, RT2316-16 was cultured in 9 different media. The media were identical to the control medium (Section 4.1), but each of the 9 formulations lacked one of the 9 components in the vitamin solution (i.e., thiamine, B1; Ca-pantothenate, B5; pyridoxine, B6-1; biotin, B7; cyanocobalamin, B12; riboflavin, B2; pyridoxamine, B6-2; nicotinic acid, B3; and *p*-aminobenzoic acid, pABA) of the control medium. Each component in the vitamin solution was included at the same concentration as in the control medium (Table S1; Supplementary Materials). The experiments included the control medium and the same medium without any of the vitamins. In terms of the mineral salts, all media of the experimental series had the same concentrations as the control medium (Table S1; Supplementary Materials).

In the second set of experiments, 4 different experiments were used to individually evaluate the effects of the three vitamers (i.e., pyridoxine (B6-1), pyridoxamine (B6-2), and pyridoxal 5′-phosphate (B6-3)) of vitamin B6. Each vitamer was used at the same concentration (0.72 mg L^−1^) and the other components in the vitamin solution (i.e., B1, B2, B5, B7, B12, B3, and pABA) and the mineral salts were included at concentrations identical to those of the control medium (Table S1; Supplementary Materials). The control medium was also included for comparison.

The third set of experiments consisted of 5 cultures that evaluated the effects of mineral salts. The salts were provided as specified volumes of S_1_ and S_2_ solutions that contained only KH_2_PO_4_ and MnCl_2_ 4H_2_O, ZnSO_4_ 7H_2_O, CoCl_2_ 6H_2_O, CuSO_4_ 5H_2_O, NiSO_4_ 6H_2_O, and FeSO_4_ 7H_2_O, respectively. The specified volumes of S_1_ and S_2_ solutions when supplied to the cultures, produced concentrations identical to those of the control medium (Table S1; Supplementary Materials). Separate cultures included a double volume of S_1_ and/or S_2_ (designated as S_1_ at 2× and S_2_ at 2×). This experimental set included a culture without the mineral salts. All the culture media contained the 9 B-vitamins at the concentration in the control medium (Table S1; Supplementary Materials).

In the last set of experiments, the effects of trehalose at two concentrations (0.5 and 1.0 g L^−1^) were evaluated. The media used in these experiments contained the 9 B-vitamins and mineral salts as specified for the control medium (Table S1; Supplementary Materials). Trehalose was included in addition to glucose as used in the control medium (Section 4.1).

In all experiments mentioned in this section, the Erlenmeyer flasks were incubated (15 ± 1 °C, orbital shaker) for 7 days (168 h). The cultures were sampled (10 mL per sample) at 24 h intervals for measurements of the biomass concentration (dry weight) and residual glucose. Microscopic inspection and plating were used to detect possible contamination. At the end of the culture period (168 h) the biomass was recovered (3220 *g*, 10 min, 4 °C) from a 50 mL sample for determination of total lipid content and fatty acid profiling of the lipids. Also, a 10 mL aliquot of each culture was used to inoculate a fresh Erlenmeyer flask (500 mL) that contained 300 mL of a sterile medium with a composition identical to the initial composition of the mother flask. These second-batch cultures were designated as subcultures. The subculture experiments were performed to evaluate any possible effect of the inoculum that in the first culture was grown in GYM (Section 4.1).

### 4.3. Bioreactor Culture

Batch cultures were carried out in a stirred tank bioreactor (Minifors 2, Infors HT, Bottmingen, Switzerland) with a 6 L vessel and a working volume of 3.8 L. Dissolved oxygen (DO) concentration was controlled to a level of 10% of the air saturation using a cascade control protocol involving stirrer speed (200–500 rpm) and subsequent control with aeration rate (1–2 L min^−1^). Incubation temperature was 15 ± 1 °C. The culture pH was not controlled. Culture samples (40 mL) were taken twice a day for measurements of the concentrations of dry biomass, glucose, amino acids, and the total lipid content of the biomass.

The inoculum for all bioreactor cultures was prepared in 500 mL Erlenmeyer flasks containing 300 mL of a sterile medium of a composition identical to the medium used in the bioreactor. This inoculum was seeded with 10 mL of the first inoculum grown in GYM (Section 4.1). Bioreactor experiments were carried out with the control medium (Section 4.1) and the CD9 medium (Table S3; Supplementary Materials). The CD9 medium was identical to the control medium, but differed in the amino acid composition; it contained 9 amino acids (aspartate, arginine, serine, glycine, threonine, alanine, proline, cysteine, and lysine).

### 4.4. Analyses

#### 4.4.1. Concentrations of Biomass and Reducing Sugars

Dry biomass concentration was determined gravimetrically. The cells were recovered by centrifugation (2057 *g*, 10 min) of a known volume of the culture, washed twice with distilled water, and dried to constant weight at 60 °C.

Glucose concentration was determined as reducing sugars using the 3,5-dinitrosalicilic acid method [43].

#### 4.4.2. Concentrations of Amino Acids

For residual amino acids, the cell-free culture supernatant was supplemented with an internal standard (10 mL of 2.5 mM α-aminobutyric acid). This sample and a standard solution of the amino acids (Waters Inc., Milford, MA, USA) were treated following the instructions in the Waters AccQ-Tag™ Ultra Derivatization Kit (Waters Inc., Milford, MA, USA). The derivatized samples were subjected to HPLC (C18, 4 μm, 3.9 × 150 mm column; Waters Inc., Milford, MA, USA). The column was maintained at 37 °C. The detection wavelength was 248 nm (Waters Inc., Milford, MA, USA). The mobile phase flow rate was 1 mL min^−1^. The mobile phase comprised AccQ-Tag Eluent (A), HPLC-grade acetonitrile (Merck) (B), and HPLC-grade water (C). A gradient elution was used as follows: 0–0.5 min 100% A; 0.5–18 min 99% A and 1% B; 18–19 min 95% A and 5% B; 19–28 min 91% A and 9% B; 28–35 min 83% A and 17% B; 35–38 min 60% B and 40% C; and 38–40 min 100% A.

#### 4.4.3. Extraction of Total Lipids and Determination of Fatty Acid Profile

Total lipids (TL) in the dry biomass were extracted using the method of Bligh and Dyer [44]. The extracted lipids were methylated and the fatty acid methyl esters were analyzed using a gas chromatograph as previously described [3].

### 4.5. Statistical Analysis

All experiments were performed in triplicate. Average values and standard deviations are reported. MATLAB R2022 (MathWorks, Inc., Natick, MA, USA) was used for one-way analysis of variance (ANOVA) and comparison of the means at the 95% confidence level.

## 5. Conclusions

A chemically defined medium (the control medium; Tables S1 and S3) with glucose as the primary carbon source and an amino acid composition based on a typical yeast extract supported good growth and lipid production of *Thraustochytrium* sp. RT2316-16. The growth rate, the final biomass concentration, the total lipids in the biomass, and the fatty acid composition were all influenced by the B-vitamins supplied in the culture media. The growth of RT2316-16 in this medium was determined by the availability of two vitamins B6 and B12. RT2316-16 preferentially consumed the following amino acids: glutamate, histidine, tyrosine, valine, methionine, isoleucine, leucine, and phenylalanine. In a well-aerated culture with the dissolved oxygen controlled to remain above 10% of the air saturation, the EPA and DHA content in the total fatty acids substantially increased (15 and 38%, respectively, after 57 h) compared to with culture in Erlenmeyer flasks. However, the biomass productivity in the bioreactor was barely half of the productivity that could be achieved in Erlenmeyer flasks, an effect ascribed to the rapid consumption of eight amino acids (36 h) and the subsequent exhaustion of the late-consumed amino acids. The reasons for this require further investigation in the future.

## Data Availability

The data supporting the findings of this study are available from the corresponding author upon reasonable request.

## Supplementary Materials

The following supporting information can be downloaded at: https://www.mdpi.com/article/10.3390/md23120482/s1, Figure S1: Time profiles of biomass (X1, X2) and glucose (S1, S2) concentrations in cultures of *Thraustochytrium* sp. RT2316-16 in the following media: (a) control medium with all vitamins; (b) control medium without (w/o) vitamins (Treatment 1 in Figure 1); (c) control medium w/o pyridoxine (B6) (Treatment 5 in Figure 1); (d) control medium w/o Ca-pantothenate (B5) (Treatment 3 in Figure 1); (e) control medium w/o thiamine (B1) (Treatment 2 in Figure 1); (f) control medium w/o biotin (B7) (Treatment 6 in Figure 1); (g) control medium w/o nicotinic acid (B3) (Treatment 4 in Figure 1); (h) control medium w/o riboflavin (B2) (Treatment 8 in Figure 1); (i) control medium w/o pABA (Treatment 10 in Figure 1); (j) control medium w/o cyanocobalamin (B12) (Treatment 7 in Figure 1); and (k) control medium w/o pyridoxamine (B6-2) (Treatment 9 in Figure 1). Filled symbols (X1, S1) are for the first culture and empty symbols (X2, S2) are for the subculture.; Figure S2. Effects of three B6 vitamers (pyridoxine, B6-1; pyridoxamine, B6-2; and pyridoxal 5′-phosphate, B6-3) on the time profiles of biomass concentration (X1, X2) and the glucose concentration (S1, S2). The culture media contained Vitamins B1, B5, B7, B12, B2, nicotinic acid, pABA and: (a) B6-1 at 0.029 mg L^−1^ and B6-2 at 0.72 mg L^−1^ (Control); (b) B6-1 at 0.72 mg L^−1^; (c) B6-2 at 0.72 mg L^−1^; and (d) B6-3 at 0.72 mg L^−1^. Filled symbols (X1, S1) are for the first culture and empty symbols (X2, S2) are for the subculture.; Figure S3. Effects of KH_2_PO_4_ (S_1_) and mineral salts (MnCl_2_ 4H_2_O, ZnSO_4_ 7H_2_O, CoCl_2_ 6H2O, CuSO_4_ 5H_2_O, NiSO_4_ 6H_2_O, and FeSO_4_ 7H_2_O) (S_2_) in the media on cultures of RT2316-16. Time profiles of biomass concentrations (X1, X2) and glucose concentrations (G1, G2) are shown in various media: (a) S_1_ and S_2_ (Control); (b) without S_1_ and S_2_ (i.e., w/o salts); (c) with S_1_ at 2× and S_2_ 2×; (d) S_1_ and S_2_ at 2×; and (e) S_1_ at 2× and S_2_. Filled symbols (X1, G1) are for the first culture and empty symbols (X2, G2) are for the subculture. Cultures with added volumes (S_1_ and S_2_) attain the concentrations of the salts as specified in Table S1. Table S1: Final concentrations of vitamins and mineral salts in the control medium.; Table S2: Compositions of the mineral salt solution, the vitamin solution I, and the vitamin solution II used in the control medium.; Table S3: Amino acid composition of the control medium and the CD9 medium.
