# Supplementary material for: Biomass Growth and Fatty Acid Production by the Marine Thraustochytrium sp. RT2316-16 in Chemically Defined Media
Source: Mar Drugs. 2025 Dec 17;23(12):482. doi: 10.3390/md23120482 (PMC12734794; doi:10.3390/md23120482)
Supplement: Supplementary file 1 [file marinedrugs-23-00482-s001.zip › marinedrugs-4010412-supplementary.pdf]

Biomass growth and fatty acid production by *Thraustochytrium* sp. RT2316-16 in chemically defined media

Liset Flores, María Paz Lefiguala, Carolina Shene

Supplemental Material

Table S1. Concentration of vitamins and mineral salts of the chemically defined media and the glucose – yeast extract medium used for the growth of *Thraustochytrium* sp. RT2316-16.

Component		mg L^{-1}	Code
Thiamine	B1	0.144	T3902
Ca-pantothenate	B5	0.072	C8731
Nicotinic acid	B3	0.072	N0761
Pyridoxine	B6	0.029	P6280
Biotin	B7	0.036	B2031*
Cyanocobalamin	B12	0.004	C3607
Riboflavin	B2	0.360	R9504
Pyridoxamine	B6	0.720	P9380
p-aminobenzoic acid	pABA	0.072	A9878
$MnCl_2 \cdot 4H_2O$		0.007	
$ZnSO_4 \cdot 7H_2O$		0.007	
$CoCl_2 \cdot 6H_2O$		0.096	
$CuSO_4 \cdot 5H_2O$		4.8	
$NiSO_4 \cdot 6H_2O$		4.8	
$FeSO_4 \cdot 7H_2O$		24	
KH_2PO_4		120	

Table S2. Composition of the mineral salt solution and the vitamin solutions (I and II) used in the control chemically defined medium (CCDM) for the growth of *Thraustochytrium* sp. RT2316-16. In the CCDM: mineral salt solution was 24 mL L^{-1}, vitamin solution I was 3.6 mL L^{-1}, and vitamin solution II was 3.6 mL L^{-1}.

Solution of mineral salts	(g L^{-1})
$MnCl_2 \cdot 4H_2O$	0.3
$ZnSO_4 \cdot 7H_2O$	0.3
$CoCl_2 \cdot 6H_2O$	0.004
$CuSO_4 \cdot 5H_2O$ $NiSO_4 \cdot 6H_2O$	0.2
$FeSO_4 \cdot 7H_2O$	0.2
KH_2PO_4	5
Solution of vitamin I	
Thiamine (B1)	0.04
Ca-pantothenate (B5)	0.02
Nicotinic acid (B3)	0.02
Pyridoxine (B6)	0.008
Solution of vitamin II	
Biotin(B7)	0.01
Cyanobalamin (B12)	0.001
Riboflavin (B2)	0.1
Pyridoxamine (B6)	0.2
p-aminobenzoic acid (pABA) (B10)	0.02

Table S3. Amino acid composition of the chemically defined medium containing 17 amino acids (CD) and the medium that contained 9 amino acids (CD9) used for the growth of *Thraustochytrium* sp. RT2316-16.

Amino acid		CD (g L^{-1})	CD9	Code
Aspartate	D	0.39	0.68	A9256
Serine	S	0.22	0.39	S4311
Glutamate	E	0.54		106445
Glycine	G	0.17	0.30	50046
Histidine	H	0.23		H8000
Arginine	R	0.42	0.74	A5006
Threonine	T	0.19	0.33	T8625
Alanine	A	0.91	1.60	A7627
Proline	P	0.19	0.33	P5607
Cysteine	C	0.02	0.04	168149
Tyrosine	Y	0.17		T8566
Valine	V	0.29		V0500
Methionine	M	0.09		M9625
Lysine	K	0.32	0.56	62840
Isoleucine	I	0.22		I2752
Leucine	L	0.36		L8000
Phenylalanine	F	0.24		P5482

	Total	5.0	5.0

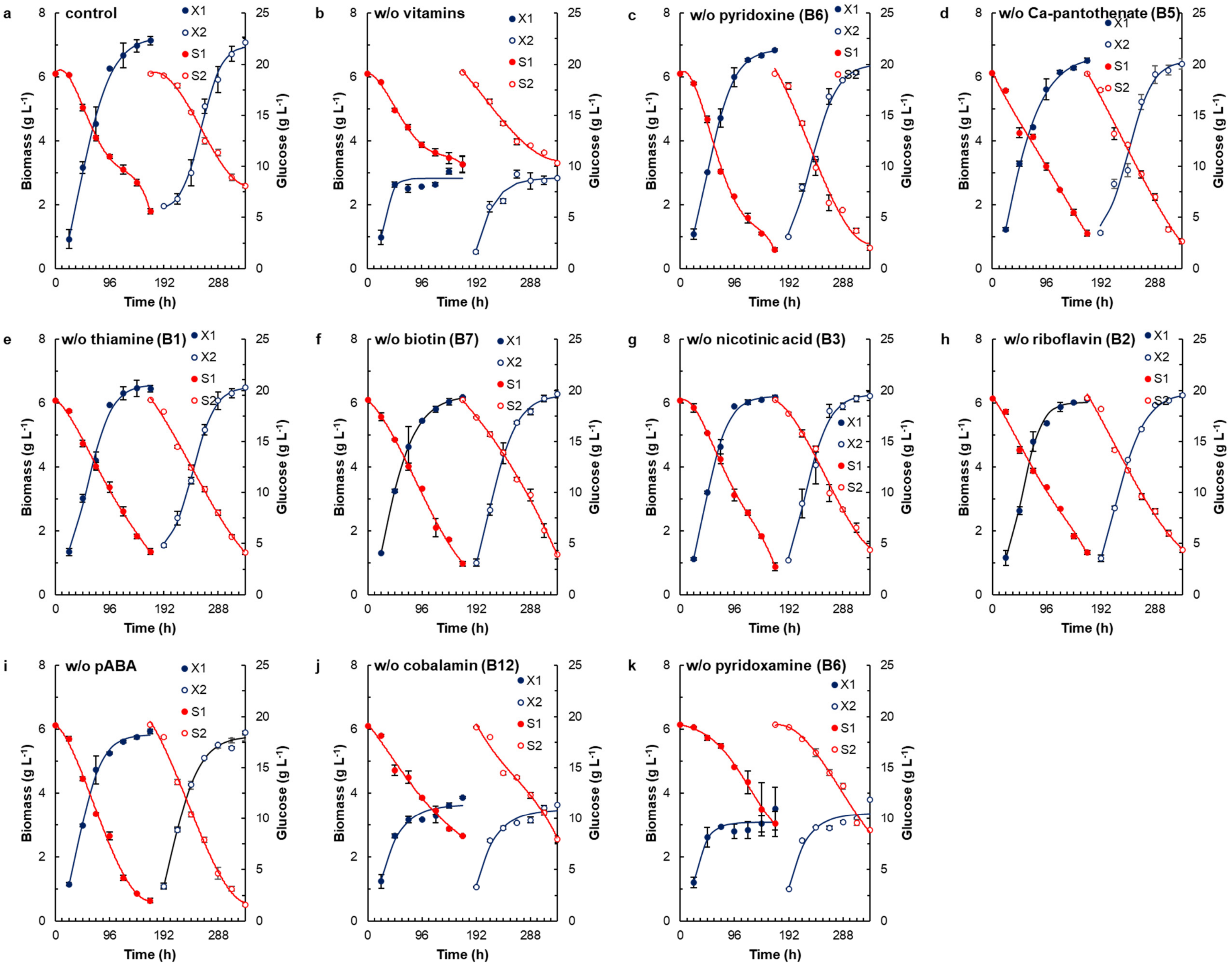

a
control
b
w/o vitamins
c
w/o pyridoxine (B6)
d
w/o Ca-pantothenate (B5)
e
w/o thiamine (B1)
f
w/o biotin (B7)
g
w/o nicotinic acid (B3)
h
w/o riboflavin (B2)
i
w/o pABA
j
w/o cobalamin (B12)
k
w/o pyridoxamine (B6)
X1
X2
S1
S2
Biomass (g L-1)
Glucose (g L-1)
Time (h)

Figure S1. Time profiles of biomass (X1 and X2) and glucose (S1 and S2) concentration in the culture of *Thraustochytrium* sp. RT2316-16 cultivated in chemically defined medium. The culture medium contained: all vitamins (a); and did not contain: vitamins (b); pyridoxine (B6) (c); Ca-pantothenate (B5) (d); thiamine (B1) (e); biotin (B7) (f); nicotinic acid (B3) (g); riboflavin (B2) (h); pABA (i); cyanocobalamin (B12) (j); or pyridoxamine (B6-2) (k).Filled symbols (X1 and S1) are experimental data in the first culture and empty symbols (X2 and S2) are data in the subculture.

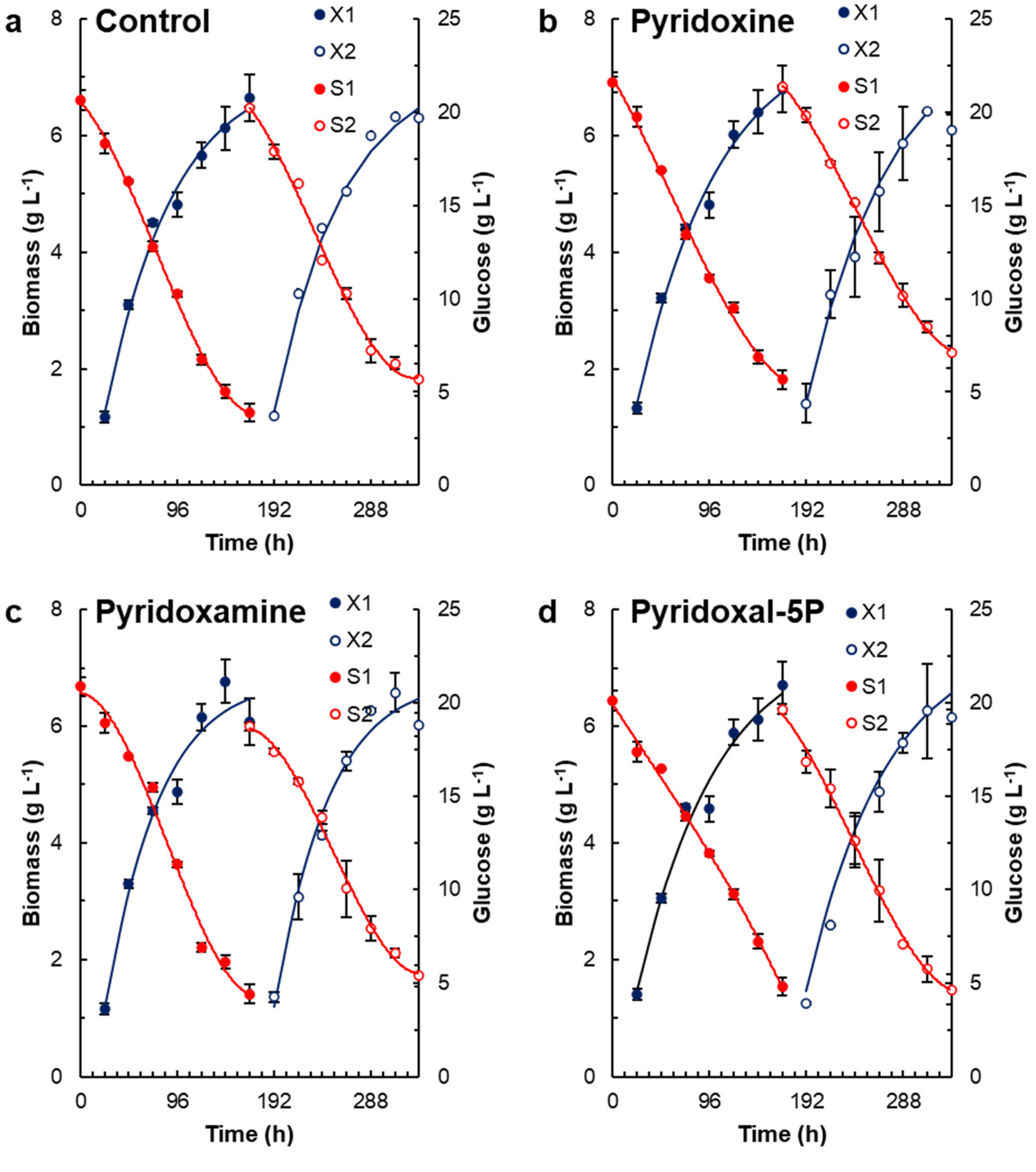

Figure S2. Effect of three B6 vitamers, pyridoxine (B6-1), pyridoxamine (B6-2) and pyridoxal 5′-phosphate (B6-3), in the chemically defined (CD) medium used to cultivate *Thraustochytrium* sp. RT2316-16 on the time profiles of biomass (X1 and X2) and glucose (S1 and S2) concentration. The culture media contained: B6-1 and B6-2 (Control) (a); only B6-1 (b); only B6-

2 (c) and only B6-3 (d). Filled symbols (X1 and S1) are experimental data in the first culture and empty symbols (X2 and S2) are data in the subculture.

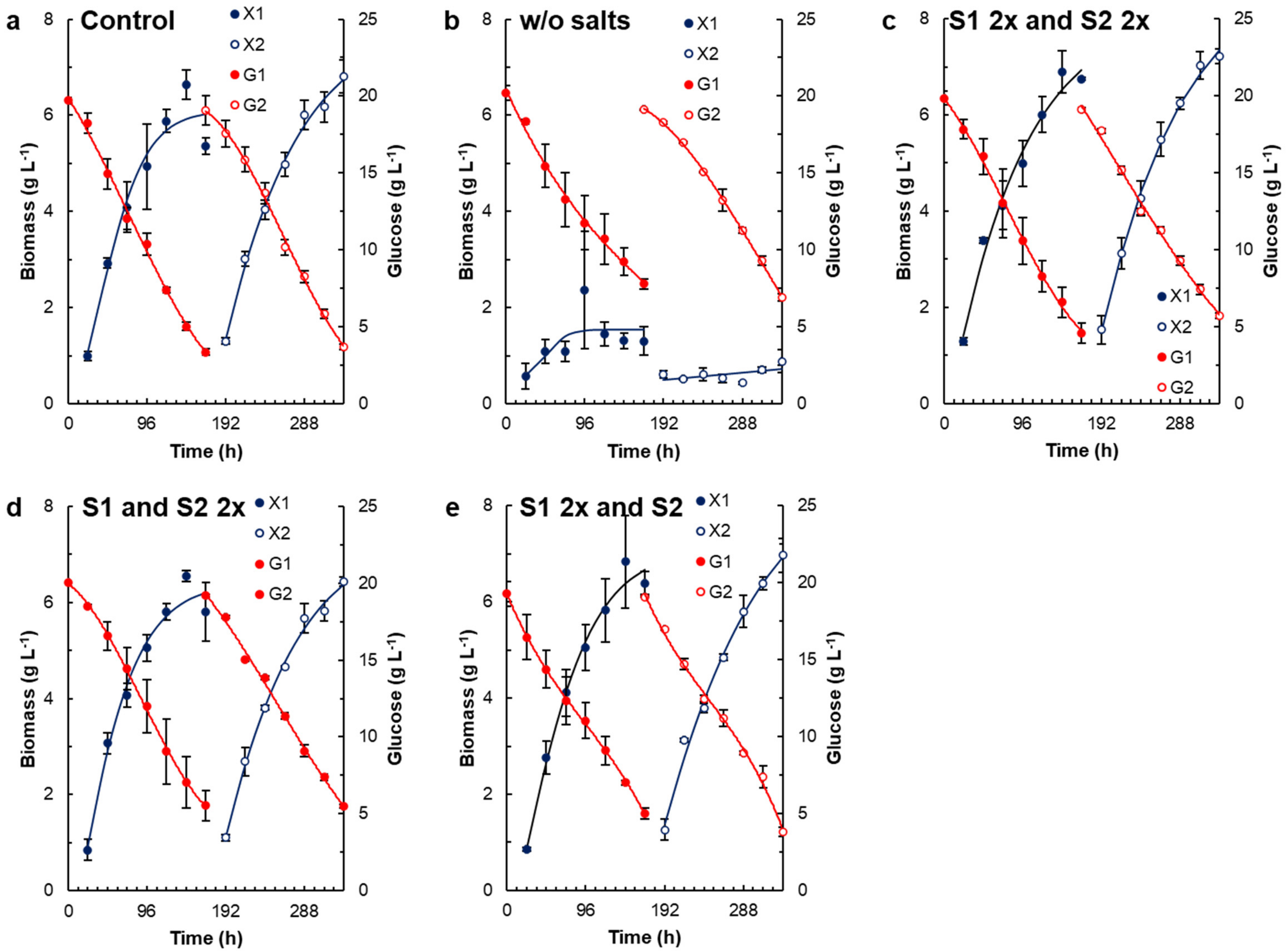
a
Control
b
w/o salts
c
S1 2x and S2 2x
d
S1 and S2 2x
e
S1 2x and S2
X1
X2
G1
G2
Biomass (g L-1)
Glucose (g L-1)
Time (h)
0
96
192
288
0
2
4
6
8
0
5
10
15
20
25

Figure S3. Effect of KH_2PO_4 (S_1) and mineral salts (S_2) in the chemically defined (CD) medium used to cultivate *Thraustochytrium* sp. RT2316-16 on the time profiles of biomass (X1 and X2) and glucose (G1 and G2) concentration. The culture media contained: S_1 and S_2 (Control) (a); without S_1 and S_2 (w/o salts) (b); S_1 at 2× and S_2 2× (c); S_1 and S_2 at 2× (d); and S_1 at 2× and S_2 (e). Filled symbols (X1 and G1) are experimental data in the first culture and empty symbols (X2 and G2) are data in the subculture.
